# YTHDC1 promotes the malignant progression of gastric cancer by promoting ROD1 translocation to the nucleus

**DOI:** 10.1007/s10565-024-09859-4

**Published:** 2024-04-04

**Authors:** Danhong Dong, Jiangpeng Wei, Weidong Wang, Haikun Zhou, Liu Hong, Gang Ji, Xisheng Yang

**Affiliations:** Department of Gastrointestinal Surgery, Xijing Hospital, Air Force Military Medical University, Xi’an, China

**Keywords:** Gastric cancer, RNA-binding proteins, N6-methyladenosine, Alternative splicing

## Abstract

RNA-binding proteins (RBPs) make vital impacts on tumor progression and are important potential targets for tumor treatment. Previous studies have shown that RBP regulator of differentiation 1 (ROD1), enriched in the nucleus, is abnormally expressed and functions as a splicing factor in tumors; however, the mechanism underlying its involvement in gastric cancer (GC) is unknown. In this study, ROD1 is found to stimulate GC cell proliferation and metastasis and is related to poor patient prognosis. In vitro experiments showed that ROD1 influences GC proliferation and metastasis through modulating the imbalance of the level of the oncogenic gene OIP5 and the tumor suppressor gene GPD1L. Further studies showed that the N6-methyladenosine (m6A) “reader” protein YTHDC1 can interact with ROD1 and regulate the balance of the expression of the downstream molecules OIP5/GPD1L by promoting the nuclear enrichment of ROD1. Therefore, YTHDC1 stimulates GC development and progression through modulating nuclear enrichment of the splicing factor ROD1.

**Graphical Abstract:**

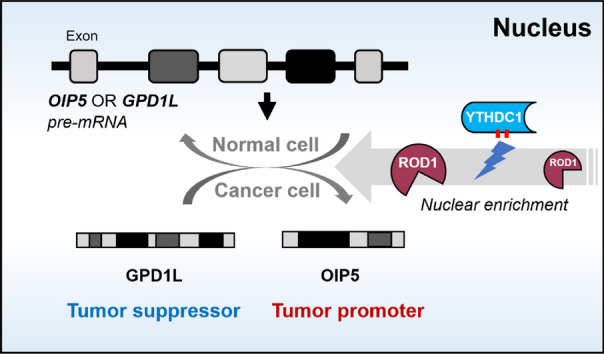

**Supplementary Information:**

The online version contains supplementary material available at 10.1007/s10565-024-09859-4.

## Introduction

Although gastric cancer (GC) exhibits decreasing morbidity and mortality trends worldwide, it is still the second main reason for cancer-related death in China (Smyth et al. 2020; Wang et al. 2021). Recently, significant progress has been made in GC treatments, including endoscopic resection, surgical resection, chemotherapy, targeted therapy, and immunotherapy. However, more than 80% of GC patients in China are in the advanced stage of disease at diagnosis, and the effect of treatment is still unsatisfactory; these factors result in poor patient prognosis (Huang et al. 2022). Studying novel molecular mechanisms underlying the occurrence of GC is of great importance for preventing and treating GC.RNA-binding proteins (RBPs) exert vital roles in the regulation of the biological functions of tumors. Previous studies have shown that RNA recruitment and binding to gene promoter regions are important mechanisms underlying RBP-mediated regulation, and these proteins can exert their regulatory effects through alternative splicing (AS), transcription, transport, storage, release, decay and other processes, which represent novel regulatory mechanisms (Papaemmanuil et al. 2011; Hopkins et al. 2016; Klingenberg et al. 2018). Regulator of differentiation 1 (ROD1, also called polypyrimidine tract-binding protein 3, PTBP3) belong to a RBP that is mainly localized to the nucleus and makes a vital role in tumorigenesis. Research has indicated that ROD1 can regulate the expression of P53 by stabilizing UBE4A, therefore stimulating the proliferation of colorectal cancer cells (Xie et al. 2022). ROD1 promotes epithelial–mesenchymal transition (EMT) by regulating TGF-β, which promotes the distant metastasis of lung cancer (Dong et al. 2022). In breast cancer, ROD1 can promote EMT by regulating ZEB1 mRNA stability (Hou et al. 2018). Moreover, studies have shown that ROD1, which is a nuclear RBP, can perform its biological functions via the AS of pre-mRNA (Sadvakassova et al. 2009; Babenko et al. 2022). In contrast, the nuclear enrichment and regulatory mechanisms of ROD1 in GC remain unknown.

N6-methyladenosine (m6A) refers to a frequently seen internal RNA modification in eukaryotes (Yan et al. 2022). Based on increasing evidence, that m6A modification regulates each RNA metabolic process, including mRNA synthesis, translation, stabilization, and splicing, and is an important mechanism underlying tumor epigenetic regulation (Oerum et al. 2021; Zhao et al. 2021; Zhang et al. 2020). Recent studies have shown that RNA splicing is generally controlled by m6A modification (Mendel et al. 2021; Goh et al. 2020; Adhikari et al. 2016). The m6A methyltransferase complex, which is consisted of METTL3, METTL14 and WTAP, participates in the transcription of RNA and the AS of genes by catalyzing m6A (Ping et al. 2014). The m6A demethylase FTO alters the RNA binding ability of the splicing regulator SRSF2 by removing the m6A modification, which in turn affects the AS of RNA (Zhao et al. 2014). By recruiting different splicing factors to the m6A site, m6A “reader” proteins can directly promote exon jumping, thus playing an important role in the interplay between trans-regulation and cis-regulation (Xiao et al. 2016). The reader protein YTH m6A RNA binding protein C1 (YTHDC1) can promote the AS of pre-mRNA by recruiting splicing factors and the nuclear output adaptor SRSF3 (Roundtree et al. 2017; Liu et al. 2020). However, the function of YTHDC1 in GC and the underlying mechanism have not been reported, and whether the nuclear enrichment of the splicing factor ROD1 is regulated by YTHDC1 needs further verification.

In this study, we discovered a novel regulatory relationship between YTHDC1 and ROD1. YTHDC1 is highly expressed in GC and promotes entry of the ROD1 protein into the nucleus, which in turn affects the imbalance of the level of the oncogenic gene OIP5 and the tumor suppressor gene GPD1L. To our knowledge, this is the first description of the mechanism underlying ROD1 protein enrichment in the nucleus.

## Materials and methods

### Patients and samples

The GC and corresponding adjacent tissue samples that were collected in this study were surgically removed from patients in Xijing Hospital during 2015–2020. Diagnosis of GC patients was in line with tissue biopsy examination. All patients did not receive chemotherapy or radiation therapy prior to surgical resection. After surgical resection, the tissue specimens were instantly frozen in liquid nitrogen. The present study gained approval from the Ethics Review Committee of Xijing Hospital.

### Animal studies

The 4–6-week-old female nude mice were applied in this study. The study was conducted according to a protocol reviewed by Animal Experiment Management Committee. After establishing a stable YTHDC1-knockdown cell line, 2 × 10^6^ cells were subject to subcutaneous injection in right side of each mouse, and the tumors were imaged and weighed after the mice were euthanized 2 weeks later. Each mouse was given injection of 2 × 10^6^ cells via tail veins in lung metastasis model, and at 4 weeks later, euthanasia was completed. Then, the lungs were removed, with metastatic nodules being counted.

### Cell culture, RNA extraction, qRT–PCR

Cell lines (GES-1, MGC803, AGS, BGC823, MKN28, MKN45, HGC27 and SGC7901 cells) were used in this study. All the cells were placed in humid incubator with 5% CO_2_ at 37 °C. The extraction of RNA was carried out using a Total RNA Kit (Omega, USA). SYBR Green Pro Taq HS kit (Agbio, China) and ABI7500 instrument were adopted for real-time fluorescence quantitative PCR. Supplementary Table 1 presents the gene-specific primer sequences.

### WB analysis

The proteins were transferred to NC membranes (Beyotime, China), and the membranes were incubated with primary antibodies against ROD1 (1:1000, # ab56918, Abcam, UK), OIP5 (1:1000, # TD12217S, Abmart, China), YTHDC1 (1:1000, # ab259990, Abcam, UK) and GPD1L (1:1000, # PS18323S, Abmart, China) at 4 °C overnight, followed by incubation with goat anti-rabbit secondary antibodies (1:2500, # EK020, ZZBIO, China) for 1 h. Then, antibody binding was identified with an ECL Ultrasensitive Chemiluminescent Solution (AccuRef Scientific, China).

### Vectors and cell transfection

A YTHDC1 knockdown lentivirus was designed, constructed and generated with hU6-MCS-Ubiquitin-EGFP-IRES-puromycin (GeneChem, China), and stably infected cell lines were selected by incubation in 2 μg/mL puromycin (MCE, China) for 5 days. The YTHDC1 shRNA sequences used were 5′-GCUGGGAGGUGUCUUUAAATT-3′ (sense) and 5′-UUUAAAGACACCUCCCAGCTT-3′ (antisense). The following oligonucleotides were synthesized (GenePharma, China): hsa-ROD1 siRNA (sense: 5′-CCAAUCACAGAGAACUUAATT-3′, antisense: 5′-UUAAGUUCUCUGUGAUUGGTT-3′); hsa-YTHDC1–1 siRNA (sense: 5′-GCUGGGAGGUGUCUUUAAATT-3′, antisense: 5′-UUUAAAGACACCUCCCAGCTT-3′); hsa-YTHDC1–2 siRNA (sense: 5′-GGAGGAAGAAGAAGAAUAUTT-3′, antisense: 5′-AUAUUCUUCUUCUUCCUCCTT-3′); hsa-OIP5–1 siRNA (sense: 5′-GGUCUUCUCCAGAGUUACA-3′, antisense: 5′-UGUAACUCUGGAGAAGACC-3′); hsa-OIP5–2 siRNA (sense: 5′-UAUCAGAGAUGGAUAUUCA-3′, antisense: 5′-UGAAUAUCCAUCUCUGAUG-3′); and hsa-OIP5–3 siRNA (sense: 5′-GCUAACGCACAAUCGCUUA-3′, antisense: 5′-UAAGCGAUUGUGCGUUAGC-3′). The GPD1L overexpression plasmid was synthesized by GenePharma (China).

### Transwell assay

In terms of the cell migration assay, the cells were adjusted to a concentration of 1 × 10^5^ cells/ml, 200 μl was supplemented to Transwell chambers (Corning, USA), and 500 μl of 1640 medium supplemented with 20 % fetal bovine serum was supplemented to the 24-well plates. After 24 h, remove the chamber from the 24-well plates, and gently remove any cells that have not passed through the chamber membrane using a cotton swab. Subsequently, the cells on the bottom of the membranes were fixed with 4% paraformaldehyde for 20 minutes and stained with crystal violet for 15 minutes. After drying, the number of cells that passed through the bottom of the chamber was counted after images were captured. Regarding the cell invasion assay, 5 × 10^5^ cells/ml were added to chambers that were precoated with Matrigel (Corning, USA), and the remaining steps remained the same as those in the migration experiment.

### Colony formation assay

After reaching 80–90% cell density, we collected cells and resuspended them in culture medium, with cell concentration being adjusted to 3 × 10^3^ cells/ml. Around 3,00 cells (100 μl) were added to each well of a 24-well plate. Following approximately 10-day culture, the culture medium was removed, and PBS slow-release solution supplemented with 0.5% crystal violet was added to each well of the 24-well plate. In addition, the staining time was approximately 30 min. After completion, cells were washed by PBS three times and observed using an inverted microscope.

### Wound-healing assay

After inoculation within the 6-well plate, cells were incubated until they reached around 90% confluence. Then, a scratch was gently made by a 200 μl pipette tip along the bottom of the 6-well plate. Images were obtained at the selected time points (0, 24 and 48 h). Finally, the degree of wound healing was analyzed with ImageJ software.

### CCK8 assay

Cells (100 μl of the 1 × 10^4^ cells/ml suspension) were supplemented into 96-well plates. After the cells attached to the wall, 10 μl of CCK8 solution (APExBIO, USA) was added to the well. 0, 24, 48, 72 and 96 h after adhesion were measured for absorbance by a microplate reader (Thermo, USA).

### EdU assay

A BeyoClick™ EdU Cell Proliferation Kit (Beyotime, China) was adopted for this experiment. The cells were incubated in 6-well plates with 1× EdU working solution at a final concentration for 2 hours. Then removed the culture medium, 1 ml of fixative was added, and the cells were fixed at room temperature for 15 minutes. The fixative was removed, with the cells being washed 3 times with washing solution. Then the cells were incubated with 1 ml of permeabilization solution per well for 10–15 minutes. Then permeabilization solution was removed, and the cells were rinsed with 1 ml of washing solution for 1–2 times. Then, the EdU assay was performed, and 0.5 ml of Click reaction solution was added to each well and incubated for 30 min in the dark. Then, the cell nuclei were stained with Hoechst. Finally, a fluorescence microscope was used for detecting the fluorescence intensity.

### Immunohistochemistry

After paraffin embedding, samples were prepared in 4-μm slices, followed by dehydration and dewaxing overnight in a drying oven at 65 °C and then completely dewaxed in xylene and gradient alcohol solutions. After high-pressure antigen repair with EDTA alkaline or sodium citrate repair solution, the sections were subjected to 12-min incubation with 30% hydrogen peroxide, and another 30-min incubation using goat serum. Then, antibodies against ROD1 (1:500, # ab56918, Abcam, UK), YTHDC1 (1:500, # ab259990, Abcam, UK), OIP5 (1:300, # TD12217S, Abmart, China), GPD1L (1:200, # PS18323S, Abmart, China), N-cadherin (1:400, # bs1172R, Bioss, China), E-cadherin (1:500, # ab40772, Abcam, UK), and vimentin (1:400, # bs0756R, Bioss, China) were supplemented for overnight incubation under 4 °C. The next day, we introduced the secondary antibody for 1-h incubation, DAB color developing solution was supplemented, and hematoxylin was used to restain the nucleus.

### Nucleoplasmic protein separation

The experiment was conducted using the Nuclear and Cytoplasmic Protein Extraction Kit (Beyotime, China). First, 200 μl of cytosolic protein extraction reagent A spiked with PMSF was supplemented to the precipitate, followed by 5 s of vigorous vortexing at maximum speed and 10–15 minutes. Then, 10 μl of cytosolic protein extraction reagent B was added and incubated for 1 minute, followed by 5 s of vigorous vortexing at maximum speed and 5 min of centrifugation at 12,000–16,000 g. The supernatant was immediately aspirated and transferred to a precooled plastic tube containing the extracted cytosolic proteins. The supernatant was immediately aspirated into a precooled plastic tube, and this supernatant was considered the extracted cytoplasmic proteins. For precipitation, the residual supernatant was fully aspirated, and 50 μl of cytosolic protein extraction reagent supplemented with the PMSF was added. Then vortexed vigorously at maximum speed for 15–30 s and later returned to the ice bath for 1 min; this process was repeated approximately 10 times. The sample was subject to centrifugation for 10 min at 4 °C. Then, the supernatant was immediately aspirated into a precooled plastic tube, and this sample was considered the extracted nucleoplasmic protein. Finally, the extracted nucleoplasmic proteins were subjected to WB.

### Statistical analysis

SPSS 27.0.1 and GraphPad Prism 8.0.1 were used for the statistical analysis. Survival analysis was caried out by the Kaplan–Meier method, and *P* values were determined by the logarithmic rank test. Differences between two independent groups were evaluated based on Student’s t test (unpaired, two-tailed). The data are indicated to be the mean ± SEM or mean ± SD, and *P* < 0.05 was though to represent a statistically significant difference. In addition, the experiments were repeated at least three times.

## Results

### ROD1 is highly expressed in GC and promotes the progression of GC cells

**Fig. 1:**
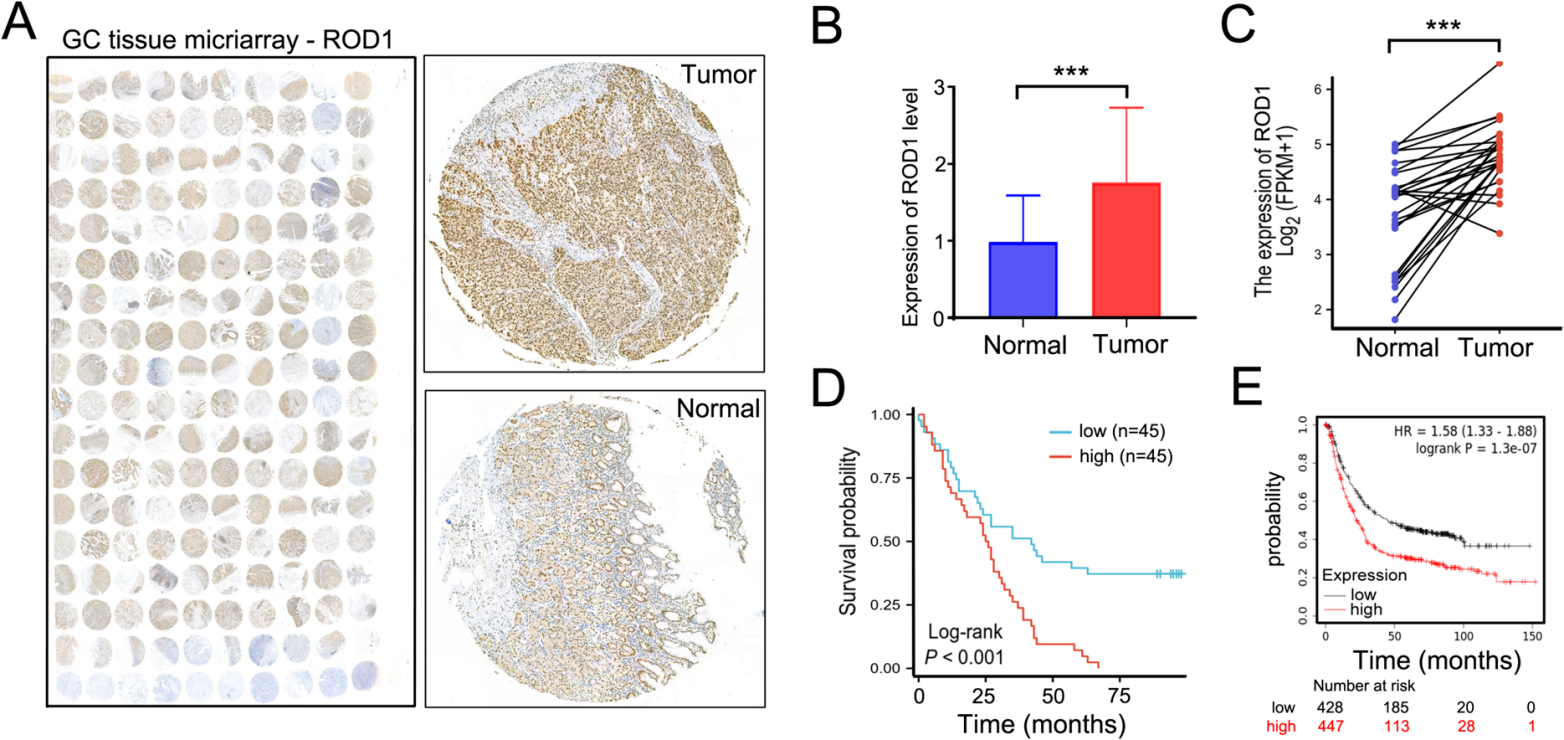
High ROD1 expression in the nucleus of GC cells is associated with poor prognosis. **A** GC tissue samples were subjected to IHC staining for ROD1; **B** IHC results showed the level of ROD1; **C** The level of ROD1 in GC was analyzed with the TCGA database (https://www.xiantaozi.com/); **D** The correlation between survival time and ROD1 level in 90 GC patients was analyzed by Kaplan–Meier; **E** The correlation between GC patients’ survival time and ROD1 was analyzed by a public database (https://kmplot.com/analysis/index.php?p=service); *** *P* < 0.001, *t* test

The mechanism by which ROD1 functions as a splicing factor in GC remains unclear. At first, we carried out immunohistochemical (IHC) staining on 90 GC tissue samples, finding that ROD1 showed up-regulation within GC tissues, mostly located in nucleus (Fig. 1A, B). In addition, as demonstrated by TCGA database analyses, ROD1 exhibited up-regulation within GC tissues (*P* < 0.001) (Fig. 1C). We divided 90 GC tissues into a high or low ROD1 expression group. Through Kaplan–Meier survival analysis, we compared the survival times of these two groups of patients, as a result, patients showing ROD1 up-regulation exhibited the decreased time of overall survival (OS) (*P* < 0.001) (Fig. 1D). Analysis of a public database (*Kaplan–Meier plotter*) revealed that the patients who had high ROD1 expression exhibited shorter OS (*P* < 0.001) (Fig. 1E).

**Fig. 2:**
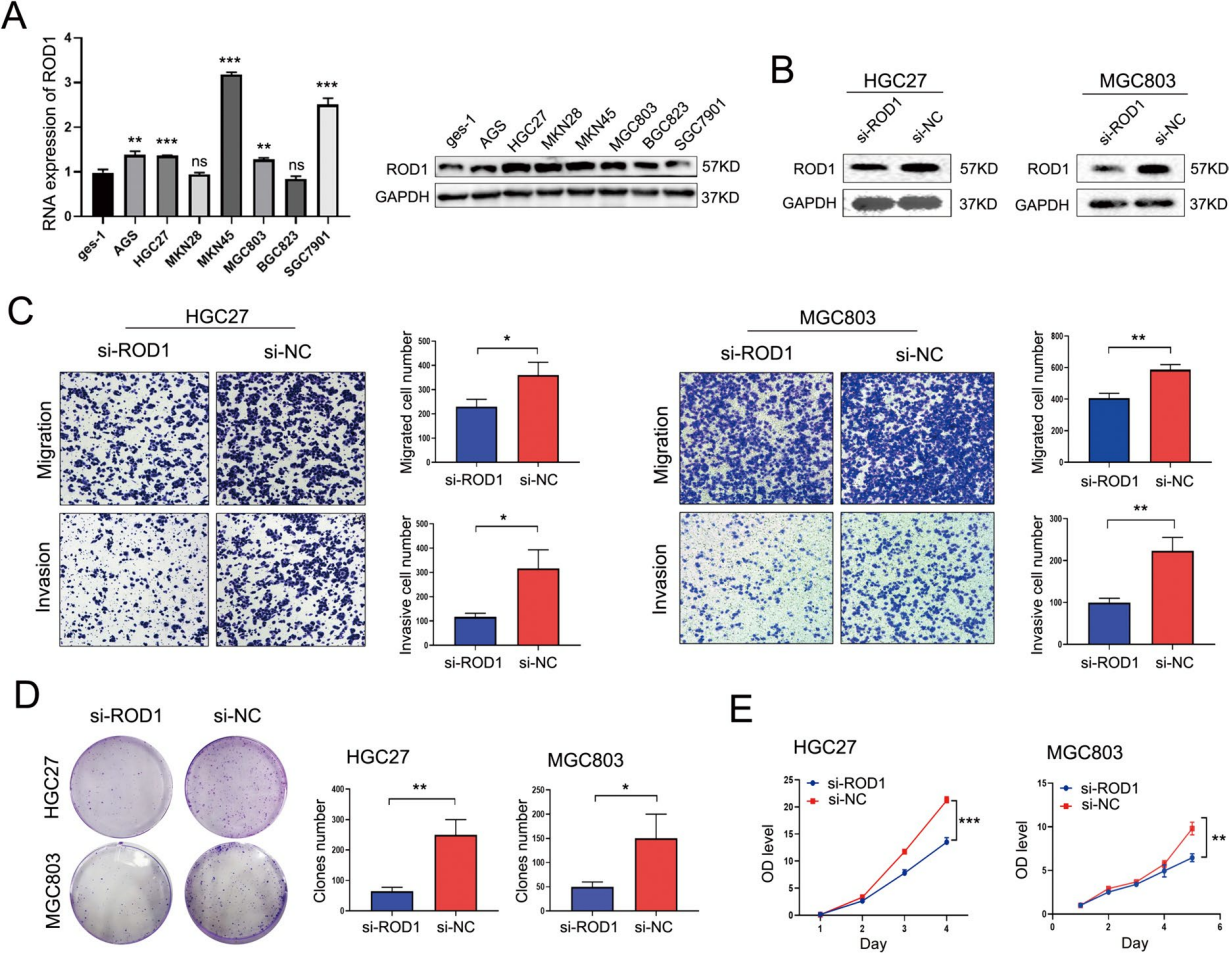
ROD1 promoted the proliferation and metastasis of GC cells. **A** ROD1 gene and protein expression levels in GC and gastric mucosal cell lines; **B** Protein level validation was performed after downregulating ROD1 in GC cell lines; **C** The changes in invasion and migration of HGC27 and MGC803 cells after the downregulation of the ROD1 gene were determined by Transwell assays (magnification × 100); **D** Plate cloning experiments and **E** Cell proliferation after downregulation of ROD1 was determined by CCK8 assays; ns, no significance; * *P* < 0.05, ** *P* < 0.01, *** *P* < 0.001, *t* test

Next, this study explored the level of ROD1 in GC cells and healthy GES-1 gastric mucosal cells by qRT–PCR and western blotting (WB) analysis, according to our findings, ROD1 showed notable up-regulation within GC cell line (Fig. 2A). Based on the ROD1 gene and protein expression levels, HGC27 and MGC803 cells were selected for use in this study. First, we confirmed the gene and protein levels of ROD1 after silencing ROD1 expression in the HGC27 and MGC803 cell lines, and the results confirmed the reliable interference efficiency of ROD1 (Supplementary Fig. 1A, Fig. 2B). By performing wound healing and Transwell assays to examine changes in GC cell migration and invasion, cell invasion and migration after transfection with siRNA targeting ROD1 (si-ROD1) was weaker than that of cells that were transfected with siRNA-NC (si-NC) (Supplementary Fig. 1B, Fig. 2C). Next, changes in cell proliferation after ROD1 interference were determined by CCK-8, EdU and plate cloning assays. In addition, the proliferation of cells in si-ROD1 group was weaker than that of cells transfected with si-NC (Supplementary Fig. 1C, Fig. 2D, E).

### ROD1 regulates the balance of OIP5/GPD1L gene expression in GC

**Fig. 3:**
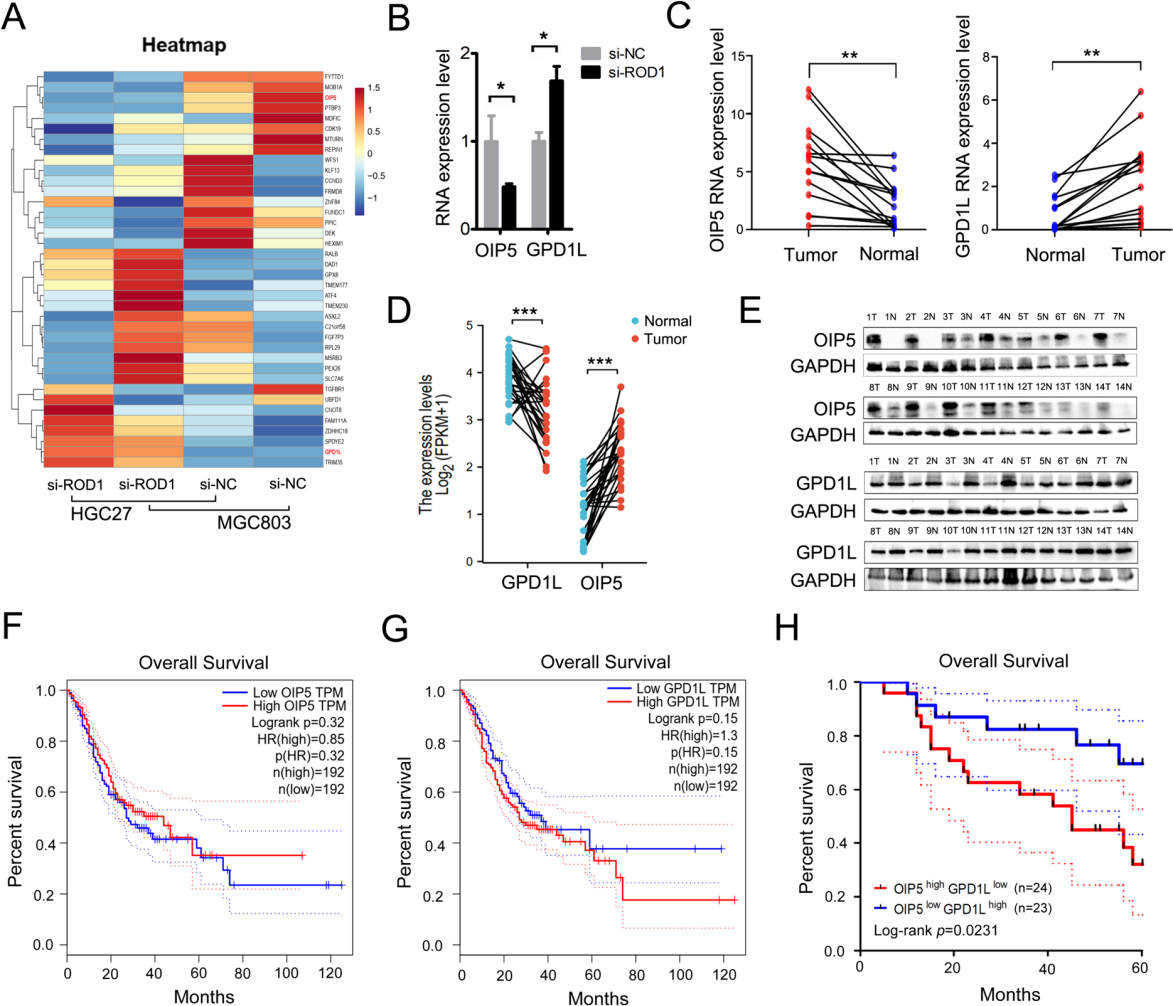
ROD1 promoted the expression of OIP5 but inhibited the expression of GPD1L. **A** After ROD1 was downregulated in GC cell lines, transcriptome sequencing was performed; **B** The level of OIP5 and GPD1L after ROD1 downregulated were measured by qRT–PCR; **C** The expression levels of OIP5 and GPD1L in GC tissues were measured by qRT–PCR; **D** The level of OIP5 and GPD1L were analyzed with the TCGA database (https://www.xiantaozi.com/); **E** The protein levels of OIP5 and GPD1L were measured by WB; **F** The correlation between survival time and the level of OIP5 and **G** GPD1L in GC patients was determined with the GEPIA database (http://gepia.cancer-pku.cn/); **H** The correlation between OS and OIP5 and GPD1L levels in GC patients was analyzed by Kaplan–Meier; * *P* < 0.05, ** *P* < 0.01, *** *P* < 0.001, *t* test

To elucidate the downstream molecules that are regulated by ROD1, we first downregulated the level of ROD1 and performed differential gene expression analysis via transcriptome sequencing in GC cell lines (Fig. 3A). Among the differentially expressed genes, we confirmed by qRT–PCR that ROD1 can promote OIP5 expression and inhibit GPD1L gene expression (Fig. 3B). We validated the gene expression levels in GC and corresponding adjacent tissues and found that the OIP5 and GPD1L genes were expressed at abnormally high and low levels in GC, respectively (Fig. 3C). The TCGA database also showed that OIP5 and GPD1L were expressed at abnormally high and low levels in GC, respectively (Fig. 3D). Subsequently, WB and IHC staining were performed to further analyze protein expression, and the results also supported the findings described above (Fig. 3E, Supplementary Fig. 2A, B). Notably, although *GEPIA* indicated that the levels of OIP5 and GPD1L were not obviously associated with OS (Fig. 3F, G), our analysis indicated that patients with OIP5^high^GPD1L^low^ tumors had shorter OS than those with OIP5^low^GPD1L^high^ tumors (*P* = 0.0231) (Fig. 3H).

### OIP5 and GPD1L play roles as oncogenes and tumor suppressors, respectively, in GC

**Fig. 4:**
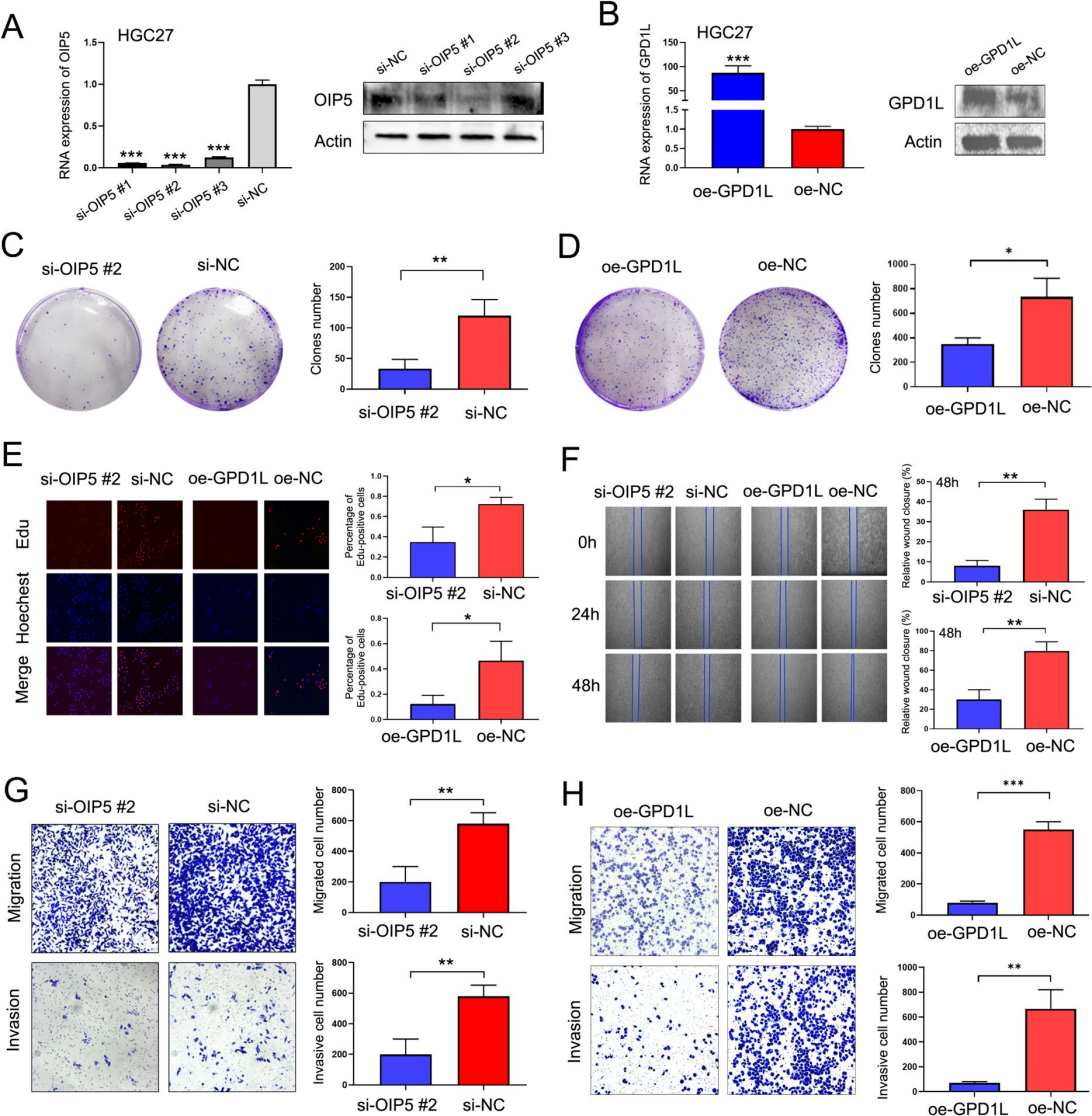
Functional analysis of OIP5 and GPD1L in GC. **A** Verification of gene and protein levels after downregulating the OIP5 gene in GC cell lines; **B** Gene and protein level validation was performed after overexpression of GPD1L; **C** Changes in proliferation after downregulating the OIP5 gene and **D** overexpressing the GPD1L gene were determined by plate cloning; **E** Cell proliferation after OIP5 silencing and GPD1L overexpression were assessed by EdU assays (magnification × 100); **F** The wound-healing assay (magnification × 40) and **G**, **H** Transwell test revealed cell invasion and migration ability after OIP5 silencing and GPD1L overexpression (magnification × 100); * *P* < 0.05, ** *P* < 0.01, *** *P* < 0.001, *t* test

Although our preliminary study demonstrated that the levels of OIP5 and GPD1L are regulated by ROD1, the functions of OIP5 and GPD1L in GC have not yet been reported. According to the high and low expression of OIP5 and GPD1L in GC, we downregulated OIP5 expression and upregulated GPD1L expression in the HGC27 cell line, and we validated the interference efficiency at the gene and protein levels (Fig. 4A, B). First, the proliferation of OIP5-knockdown and GPD1L-overexpressing cells was analyzed by plate cloning formation and EdU assays, respectively. The findings demonstrated that cell proliferation of si-OIP5 group notably decreased relative to si-NC group (Fig. 4C, E). Moreover, the proliferation of oe-GPD1L cells transfected with the GPD1L overexpression plasmid decreased (Fig. 4D, E). Then, we further proved that cell migration and invasion of si-OIP5 and oe-GPD1L groups were weaker than those in the control group as shown by wound-healing and Transwell experiments (Fig. 4F, G, H).

### The m6A reader protein YTHDC1 is highly expressed in GC and can promote cell proliferation and metastasis

**Fig. 5:**
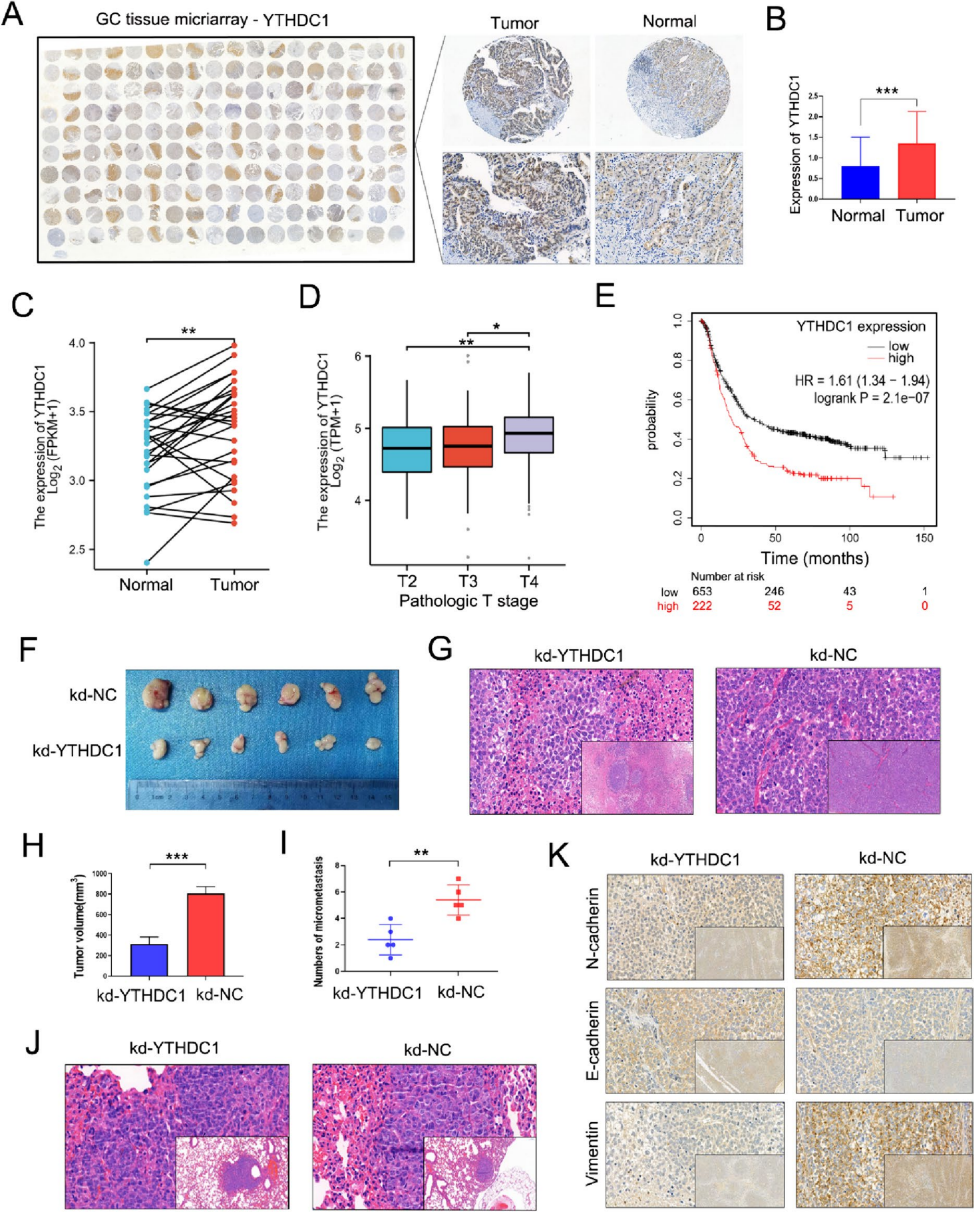
YTHDC1 is highly expressed in GC and can promote tumor proliferation and metastasis in vivo. **A**, **B** GC tissues were subjected to IHC staining for YTHDC1 protein, the results were calculated (magnification × 100); **C** The TCGA database (https://www.xiantaozi.com/) was used to analyze the YTHDC1 level and **D** the correlation between YTHDC1 and pathological stage of GC; **E** Database analysis (https://kmplot.com/analysis/index.php?p=service) of the relationship between survival time and YTHDC1 expression level in patients with GC; **F** Tumors were examined; **G** HE staining of subcutaneous tumors (magnification × 100 and × 400); **H** Tumor weight was measured; **I** The number of lung metastases was measured; **J** HE staining of lung metastases (magnification × 100 and × 400); **K** IHC staining showed the level of N-cadherin, E-cadherin and Vimentin in subcutaneous tumors (magnification × 100 and × 400); * *P* < 0.05, ** *P* < 0.01, *** *P* < 0.001, *t* test

Previous studies have confirmed that ROD1 is an RBP that can regulate splicing enzyme activity (Tan et al. 2015). Recent studies have demonstrated that the m6A “reader” protein YTHDC1 can participate in the regulation of pre-mRNA by AS, but the role of YTHDC1 in GC has not been reported. We further performed IHC staining on 90 GC tissue specimens and found that YTHDC1 expression markedly increased within GC tissues, especially in nucleus (Fig. 5A, B). The TCGA database also indicated abnormally high YTHDC1 expression in GC (Fig. 5C), which was associated with the pathological stage of GC (Fig. 5D). Additionally, based on analyses using Kaplan–Meier plotter database, GC patients showing YTHDC1 up-regulation exhibited the shorter OS (*P* < 0.001) (Fig. 5E). To further demonstrate effect of YTHDC1 on GC malignant progression, we first established the nude mouse model of subcutaneous tumor. Human MGC803 GC cells were transfected with a lentiviral vector to knock down YTHDC1 expression (kd-YTHDC1), and added to a matrix gel, prior to subcutaneous injection in nude mouse skin; then, subcutaneous tumor generation was found 2 weeks later. Following sampling, the mouse tumor weights of kd-YTHDC1 group notably decreased relative to kd-NC group (Fig. 5F, H). Representative hematoxylin and eosin (HE) staining demonstrated the obtained results (Fig. 5G). Similarly, we established lung metastasis models through injection of cancer cells in mouse tail veins. According to these results, the lung metastasis number of the kd-YTHDC1 group was also notably lower when relative to control group after 1 month (Fig. 5I, J). IHC staining of subcutaneous tumor tissues from mice previously downregulated by YTHDC1 suggested that YTHDC1 may promote EMT (Fig. 5K, Supplementary Fig. 3A).

**Fig. 6:**
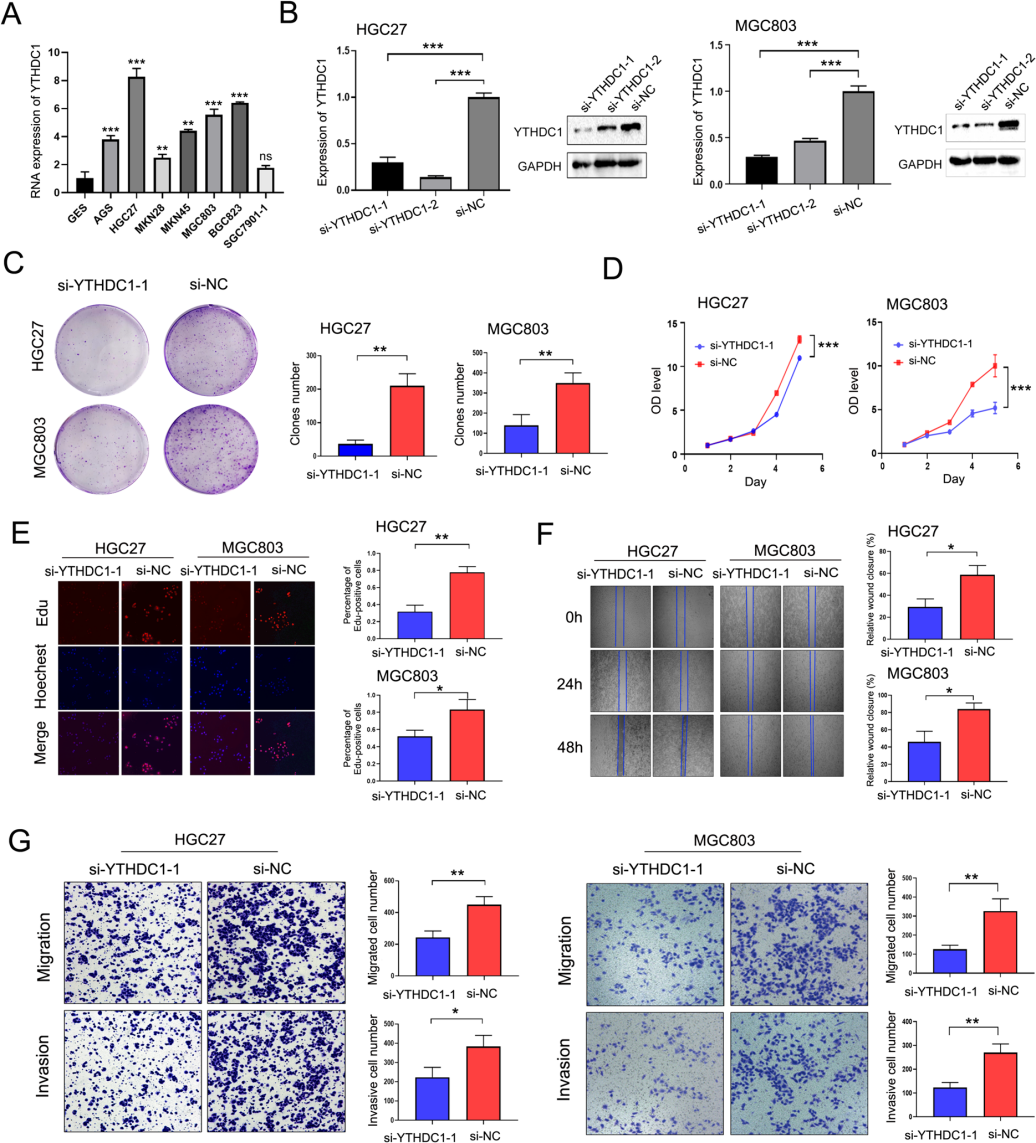
Function of YTHDC1 in vitro. **A** The YTHDC1 gene level in GC and normal gastric mucosa cell lines were measured by qRT–PCR; **B** Gene and protein levels were verified after YTHDC1 was downregulated in HGC27 and MGC803 cells; **C** Plate cloning assay, **D** CCK8 and **E** EdU assays were used to assess changes in cell proliferation after YTHDC1 gene silencing (magnification × 100); **F** Wound-healing (magnification× 40) and **G** Transwell assays were used to assess changes in migration and invasion of cells after YTHDC1 gene silencing; ns, no significance (magnification × 100); * *P* < 0.05, ** *P* < 0.01, *** *P* < 0.001, *t* test

Next, after analyzing YTHDC1 levels within GC cells and normal gastric mucosal cells, we selected HGC27 and MGC803 cells for further study and confirmed their expression at the gene and protein levels after YTHDC1 gene silencing; the results confirmed the reliability of the interference efficiency (Fig. 6A, B). We analyzed changes in cell proliferation after YTHDC1 gene silencing through plate cloning, CCK-8 and EdU assays. According to our findings, the proliferation of HGC27 and MGC803 cells of si-YTHDC1 group decreased relative to si-NC- transfected group (Fig. 6C, D, E). Moreover, through wound-healing and Transwell assays, cell metastasis ability of si-YTHDC1 group further decreased (Fig. 6F, G).

### YTHDC1 can promote the nuclear enrichment of ROD1 and disrupt the balance of OIP5/GPD1L expression

**Fig. 7:**
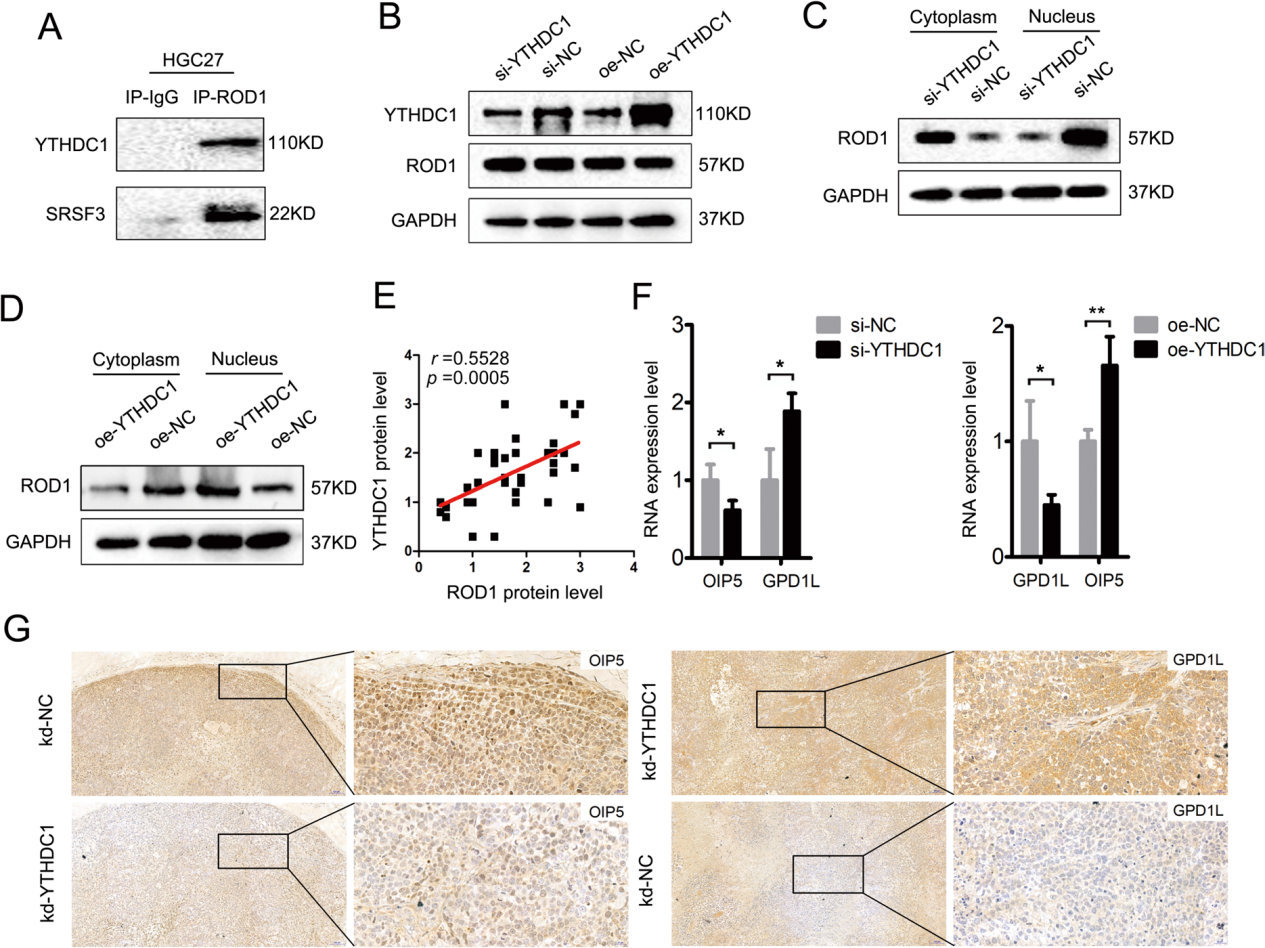
YTHDC1 functions by promoting entry of the ROD1 protein into the nucleus. **A** Co-IP was used to assess the interaction between the ROD1 protein and YTHDC1/SRSF3 protein; **B** WB was used to detect the ROD1 protein in whole-cell lysates after YTHDC1 expression interference; **C** After YTHDC1 downregulation, the changes in ROD1 protein expression in the nucleus and cytoplasm of HGC27 cells were determined by WB; **D** After YTHDC1 was upregulated, the changes in ROD1 protein expression in the nucleus and cytoplasm were determined by WB; **E** The correlation between YTHDC1 expression and ROD1 expression in the nucleus was analyzed by IHC; **F** The level of OIP5 and GPD1L were measured after YTHDC1 silencing or overexpression by qRT–PCR; **G** IHC staining of OIP5 and GPD1L proteins in subcutaneous tumors (magnification × 100 and × 400); * *P* < 0.05, ** *P* < 0.01, *t* test

**Fig. 8:**
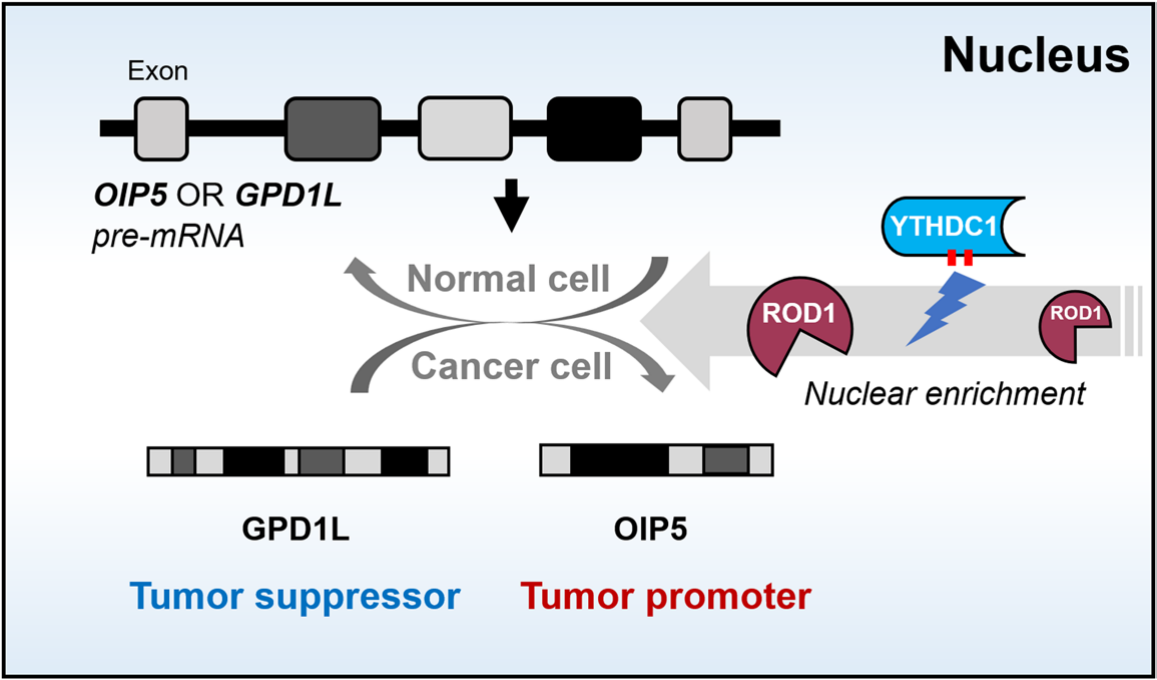
Model by which YTHDC1 regulates GC cell malignant growth and metastasis

It has been recently indicated that YTHDC1 can promote the AS of pre-mRNAs by recruiting splicing factors and the nuclear output adaptor SRSF3 (Roundtree et al. 2017; Liu et al. 2020). To elucidate whether the imbalance of ROD1-mediated regulation of OIP5 and GPD1L expression in GC is controlled by YTHDC1, we conducted further research. Interestingly, co-IP experiments revealed that ROD1 interacts with the YTHDC1/SRSF3 proteins (Fig. 7A). To further elucidate the regulatory relationship between YTHDC1 and ROD1, we silenced or upregulated YTHDC1 gene expression in the HGC27 cell line and observed that there existed no obvious change in ROD1 protein levels in whole-cell lysates (Fig. 7B). Considering that ROD1 functions mainly as a splicing factor in the nucleus, we separated the nuclear and cytoplasmic fractions of YTHDC1-knockdown HGC27 cells and discovered that ROD1 protein levels were decreased in the nucleus but significantly elevated in the cytoplasm (Fig. 7C). Similarly, when we overexpressed YTHDC1, ROD1 protein expression was increased in the nucleus but significantly decreased in the cytoplasm (Fig. 7D). By analyzing the expression of YTHDC1 and ROD1 in the tissue array, we found that the YTHDC1 and ROD1 levels in tumor nuclei were positively correlated (Fig. 7E). Next, we observed changes in the expression levels of OIP5 and GPD1L in YTHDC-knockdown or YTHDC1-overexpressing HGC27 cell lines and found that YTHDC1 promoted the levels of OIP5 or inhibited the expression of GPD1L (Fig. 7F). Moreover, IHC staining was performed on the subcutaneous tumor tissues of mice in which YTHDC1 was downregulated, and it was found that OIP5 expression was lower in the kd-YTHDC1 group, while GPD1L expression was increased (Fig. 7G, Supplementary Fig. 3B). Therefore, YTHDC1 was shown to affect the malignant progression of GC by promoting the nuclear enrichment of ROD1 (Fig. 8).

## Discussion

Abnormally expressed RBPs can regulate gene expression through transcriptional and posttranscriptional processes and thus participate in the malignant proliferation, metastasis, apoptosis and drug resistance of tumor cells. Although mutations and changes in the copy numbers of RBPs account for only 15.2% of total RBPs, dysregulation of RBP expression has been observed in many tumors (Sebestyén et al. 2016). ROD1 is a type of RBP that is mainly localized to the nucleus. It has been previously indicated that ROD1 is engaged in mediating the progression of various cancers. For example, ROD1 can bind to IRES region in HIF-1α mRNA and participates in colorectal cancer growth and invasion through activating HIF-1α translation (Hou et al. 2019). miR-297 can hinder PI3K/AKT pathway by downregulating ROD1, which can therefore inhibit HCC cell growth, invasion and migration (Lu et al. 2023). According to our results, ROD1 can promote GC cell proliferation and metastasis and is related to poor prognosis in patients undergoing GC, but the regulatory mechanism of ROD1 in GC needs further exploration.

Previous studies suggested that approximately 5% of human genes are involved in AS, but with the further development of human genome sequencing, it was found that at least 92–94% of human genes are involved in AS (Wang et al. 2008). The different splice variants that are produced by AS perform different biological functions, and an imbalance in splicing that leads to the abnormal expression of splice variants can promote disease genesis and progression (Mirtschink et al. 2015). ROD1, which is the splicing regulator, plays a vital regulatory role in cancer progression. Notably, splicing factors in tumors not only generate splice variants of different lengths via the AS of pre-mRNAs but also generate mRNAs from pre-mRNAs. In GC, we discovered that abnormally high ROD1 expression affects the malignant progression of tumors by promoting an imbalance in the expression the downstream molecular oncogene OIP5 and the tumor suppressor gene GPD1L. From the perspective of molecular function, these two genes can modulate GC cell growth and invasion in an independent manner. However, from the perspective of survival time, only the simultaneous abnormal expression of both can predict poor prognosis in GC patients, and the two seem to be interdependent. Whether ROD1 is involved in the regulation of OIP5 and GPD1L production through AS needs to be further verified in subsequent studies. However, ROD1, which is a splicing regulator, mainly plays a transcriptional regulatory role in the nucleus, but the mechanism underlying ROD1 enrichment in the nucleus is still unclear; this mechanism is an important research focus of this study.

Recently, increasing evidence has revealed the role of m6A modifications in regulating AS. Studies have shown that during AS, m6A-modified exons tend to remain in mRNAs after splicing (Xiao et al. 2016; Azzam et al. 2022). M6A modifications affect the regulation of natural splicing primarily by binding to RBPs or by directly influencing the interaction between RNAs and RBPs (Zhu et al. 2023). M6A “reader” proteins can participate in regulating splicing by recruiting different splicing factors to m6A sites. There is evidence that the m6A “reader” protein YTHDC1 promotes exon inclusion in target mRNAs through the recruitment of pre-mRNA splicing factor SRSF3 but suppressing the binding of SRSF10 to mRNA (Xiao et al. 2016). The competitive binding of SRSF3 and SRSF10 indicated that YTHDC1 can regulate mRNA splicing through pre-mRNA splicing factor recruitment and regulation. However, the mechanism by which YTHDC1 functions in GC is not been illustrated. Interestingly, YTHDC1 shows high expression within GC, which can facilitate the malignant progression of GC cells, which is related to poor patient prognosis. To elucidate whether the mechanism underlying the function of ROD1 in GC is regulated by YTHDC1, we conducted a further study. Notably, through co-IP experiments, we demonstrated that YTHDC1 can interact with ROD1/SRSF3 proteins. Further analysis of protein levels after nucleoplasmic separation revealed that although YTHDC1 could not regulate the expression of ROD1, it could promote the nuclear translocation of the ROD1 protein and participate in regulating downstream molecules OIP5 and GPD1L. As the mechanism by which ROD1 is highly expressed in GC remains unclear, we were unable to conduct rescue experiments to further validate this pathway, and its detailed underlying mechanisms will be further investigated in future studies. Therefore, this study elucidates the regulatory mechanism of ROD1 entry and postentry into the nucleus.

In summary, this study suggested that YTHDC1 functions as a key factor in the nuclear enrichment of ROD1, which in turn affects the level of the oncogenic gene OIP5 and the tumor suppressor gene GPD1L; this work study offers novel strategies for preventing and treating GC.

## Supplementary information

Supplementary file 1 (ZIP 9651 kb)

## Data Availability

No datasets were generated or analysed during the current study.

## Abbreviations

AS: Alternative splicing
EMT: Epithelial–mesenchymal transition
GC: Gastric cancer
HE: Hematoxylin and eosin
IHC: Immunohistochemical
m6A: N6-methyladenosine
OS: Overall survival
PTBP3: Polypyrimidine tract-binding protein 3
RBPs: RNA binding proteins
ROD1: Regulator of differentiation 1
YTHDC1: YTH m6A RNA binding protein C1.
