# Supplementary material for: YTHDC1 promotes the malignant progression of gastric cancer by promoting ROD1 translocation to the nucleus
Source: Cell Biol Toxicol. 2024 Apr 4;40(1):19. doi: 10.1007/s10565-024-09859-4 (PMC10995098; doi:10.1007/s10565-024-09859-4)
Supplement: Supplementary file 1 — (ZIP 9651 kb) [file 10565_2024_9859_MOESM1_ESM.zip › Supplementary materials.docx]

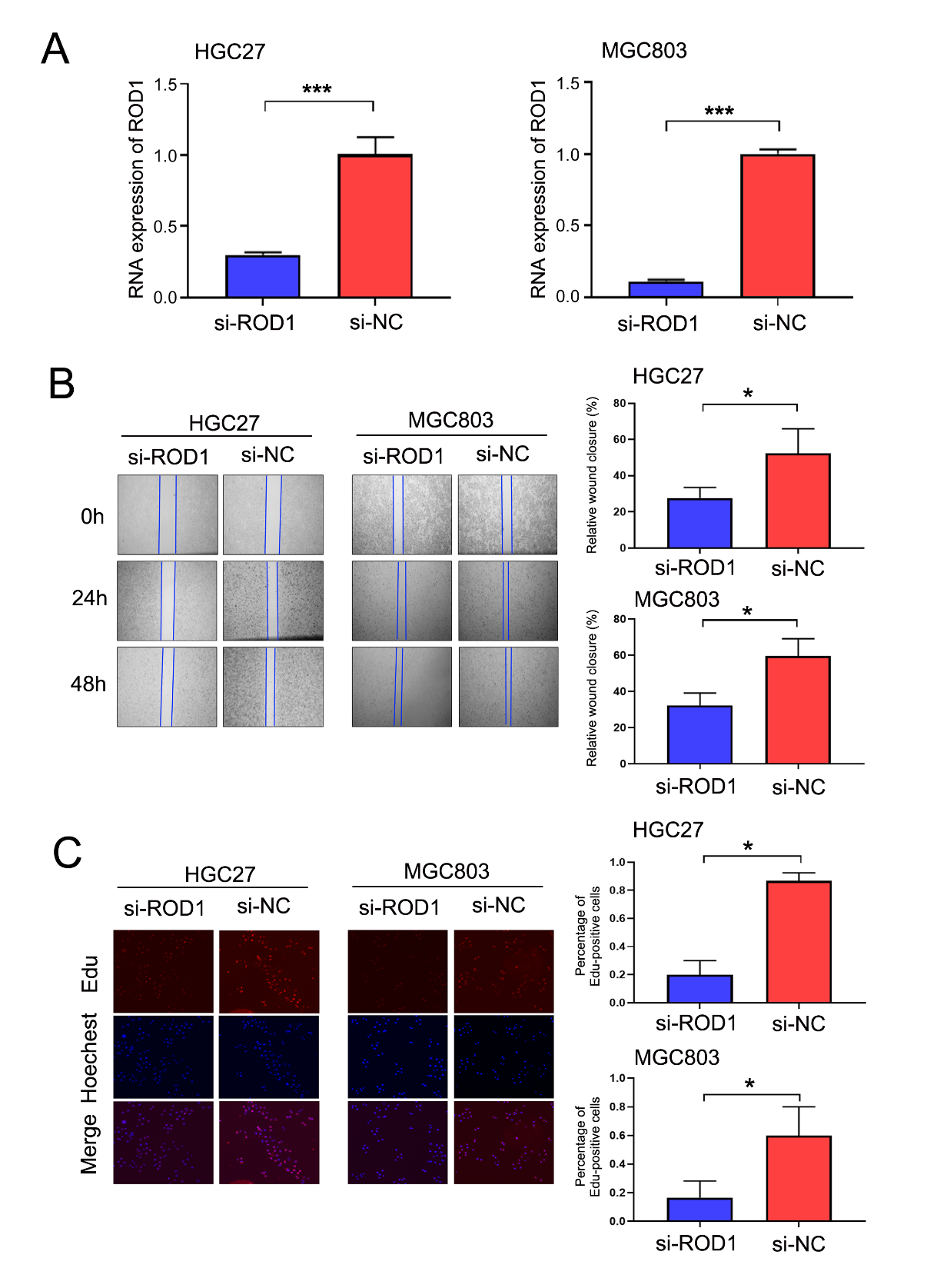


**Supplementary Figure 1. ROD1 promoted the progression of GC cells.** (A) Verification of gene expression after the downregulation of ROD1 in GC cell lines; (B) Changes in cell migration after ROD1 gene downregulation in HGC27 and MGC803 cells were determined by a wound healing assay (magnification ×40); (C) The cell proliferation after the downregulation of ROD1 was assessed by EdU assay (magnification ×100); *** *P*<0.001, t test.


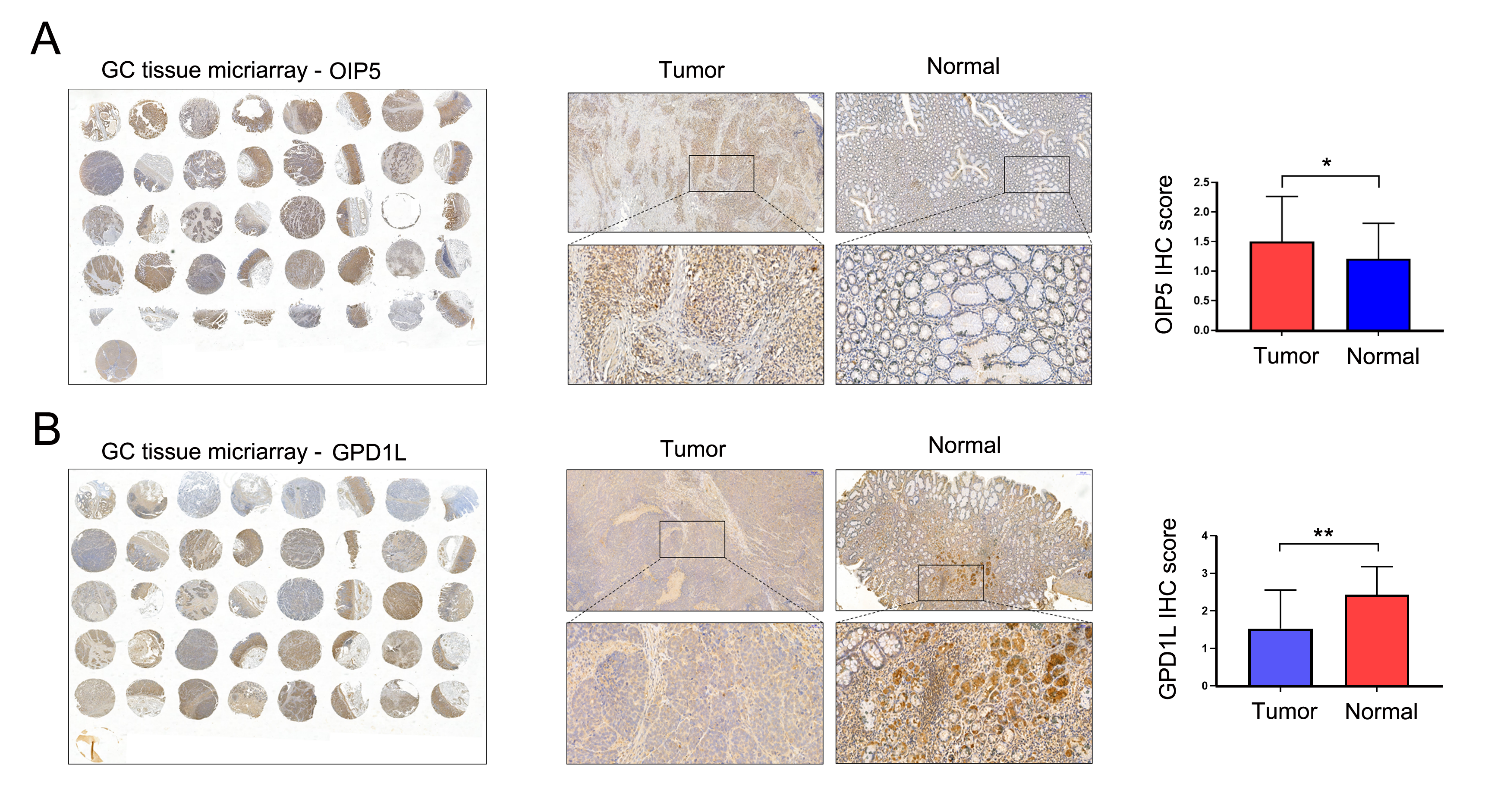


**Supplementary Figure 2. ROD1 promoted the expression of OIP5 but inhibited the expression of GPD1L.** (A) IHC staining for the OIP5 protein was performed in GC tissue samples, and the results were statistically analyzed (magnification ×40 and ×200); (B) IHC staining for the GPD1L protein was performed in GC tissue samples, and the results were statistically analyzed (magnification ×40 and ×200); * *P*<0.05, ** *P*<0.01, t test.


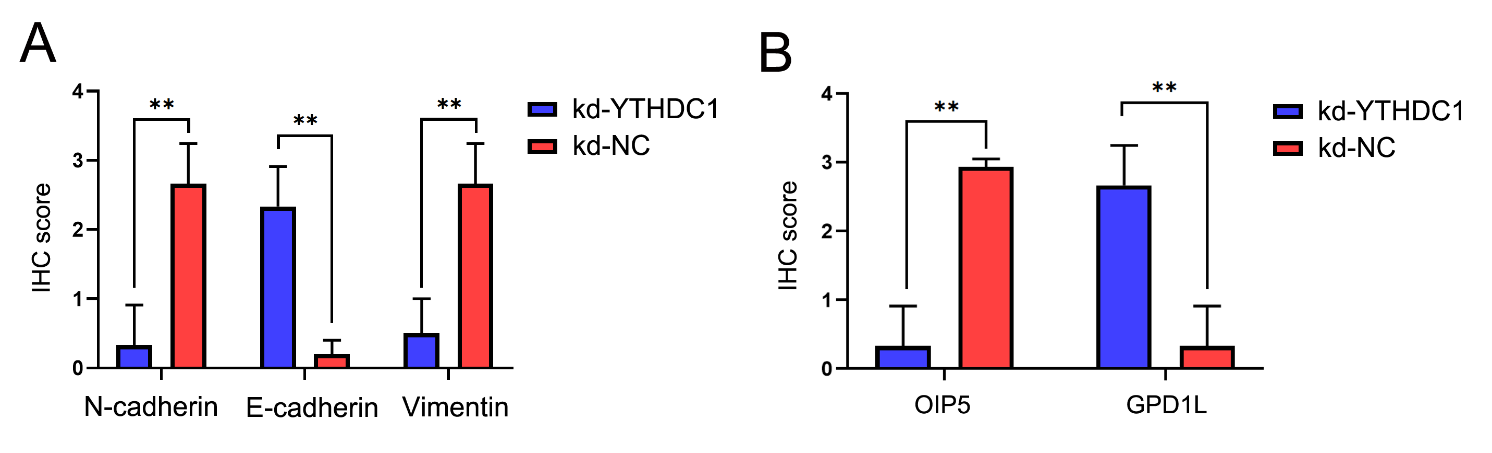


**Supplementary Figure 3.** IHC staining revealed the level changes of (A) EMT markers_,_ (B) OIP5 and GPD1L in subcutaneous tumors after YTHDC1 knock down; ** *P*<0.01, t test.

**Supplementary Table 1**

**Gene-specific primers**

| **name** | **Gene ID in NCBI** | **Sequence** |
| --- | --- | --- |
| ROD1-F | 9991 | 5'-GCTGTCAGTGCCGTCCAAT-3' |
| ROD1-R |  | 5'-AGGGTAACAGGGTAAAAGAGGT-3' |
| YTHDC1-F | 91746 | 5'-TTCCTCCCAGTGTCCATGTC-3' |
| YTHDC1-R |  | 5'-GGATTACGGCTGGCTCCAAA-3' |
| OIP5-F | 11339 | 5'-TCACTCAAAGGCAGCTATCTC-3' |
| OIP5-R |  | 5'-TTTTCTGGCTTGGACTGGTCA-3' |
| GPD1L-F | 23171 | 5'-GCTGGGAATCACCCTCATCAA-3' |
| GPD1L-R |  | 5'-TCTCCATTACTTTGCTGCCGAT-3' |
