# Supplementary figures and images for: YTHDC1 promotes the malignant progression of gastric cancer by promoting ROD1 translocation to the nucleus
Source: Cell Biol Toxicol. 2024 Apr 4;40(1):19. doi: 10.1007/s10565-024-09859-4 (PMC10995098; doi:10.1007/s10565-024-09859-4)

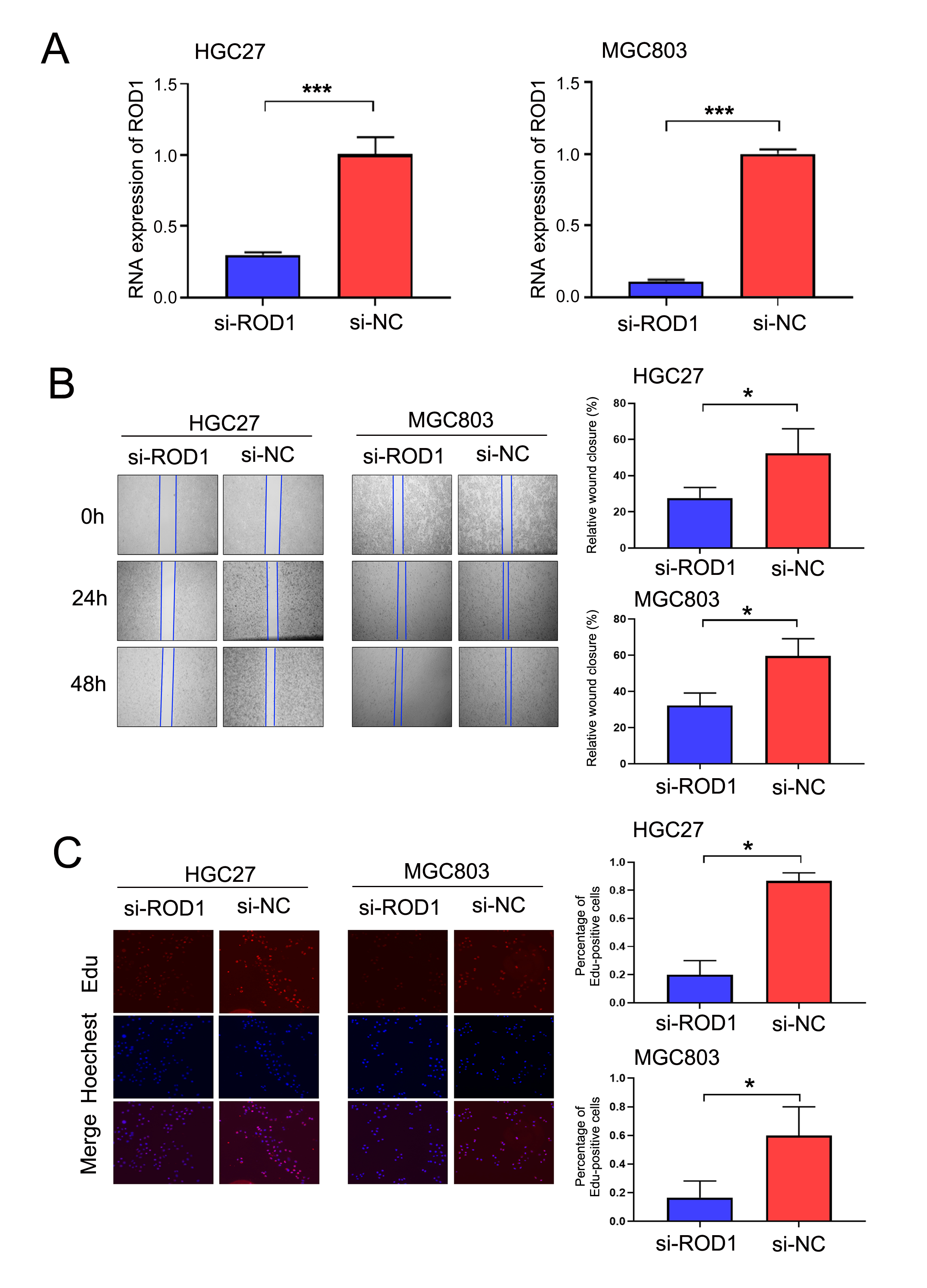

Supplement: Supplementary file 1 — (ZIP 9651 kb) [file 10565_2024_9859_MOESM1_ESM.zip › Supplemental Figure 1.tif]

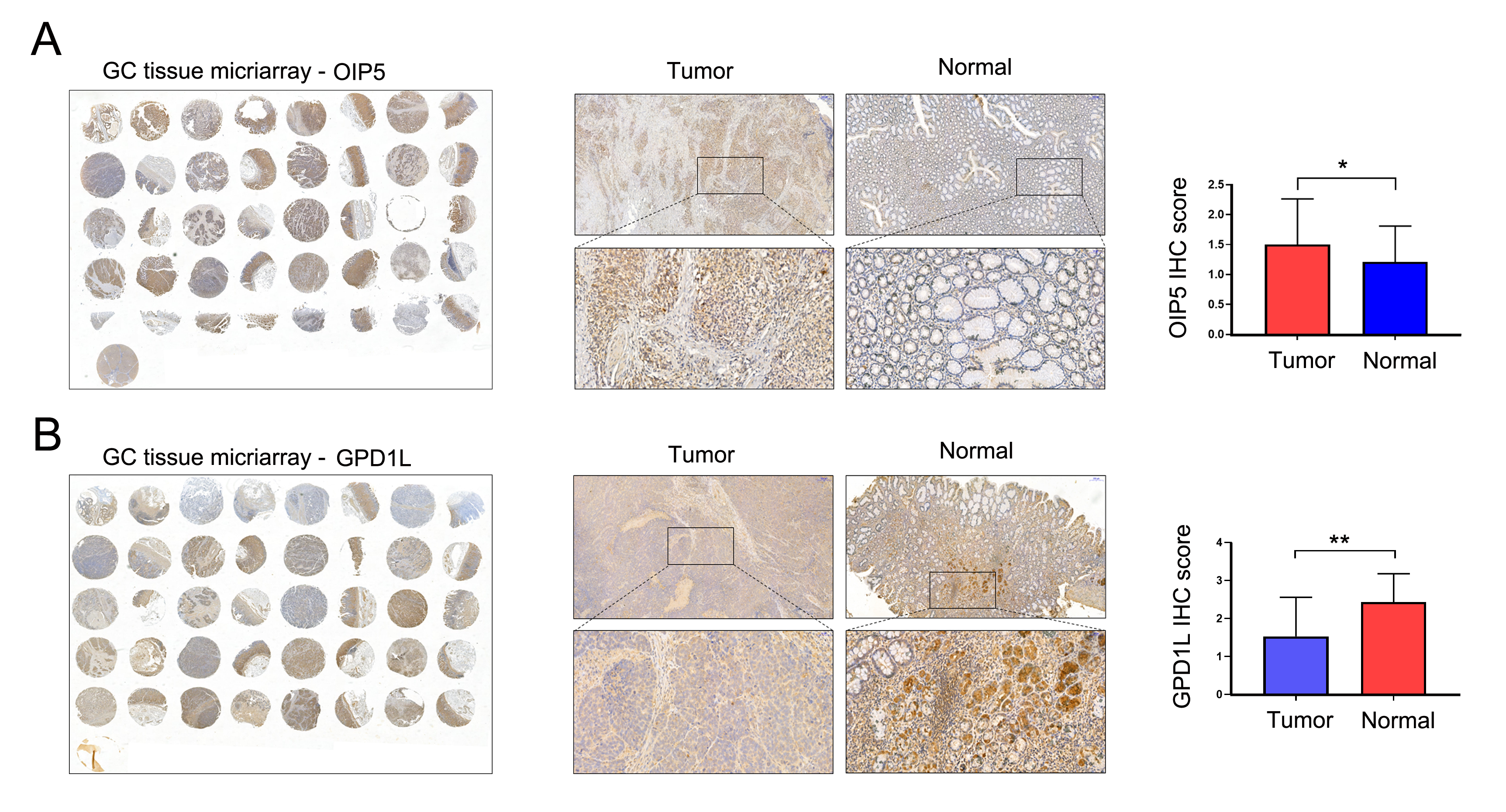

Supplement: Supplementary file 1 — (ZIP 9651 kb) [file 10565_2024_9859_MOESM1_ESM.zip › Supplemental Figure 2.tif]

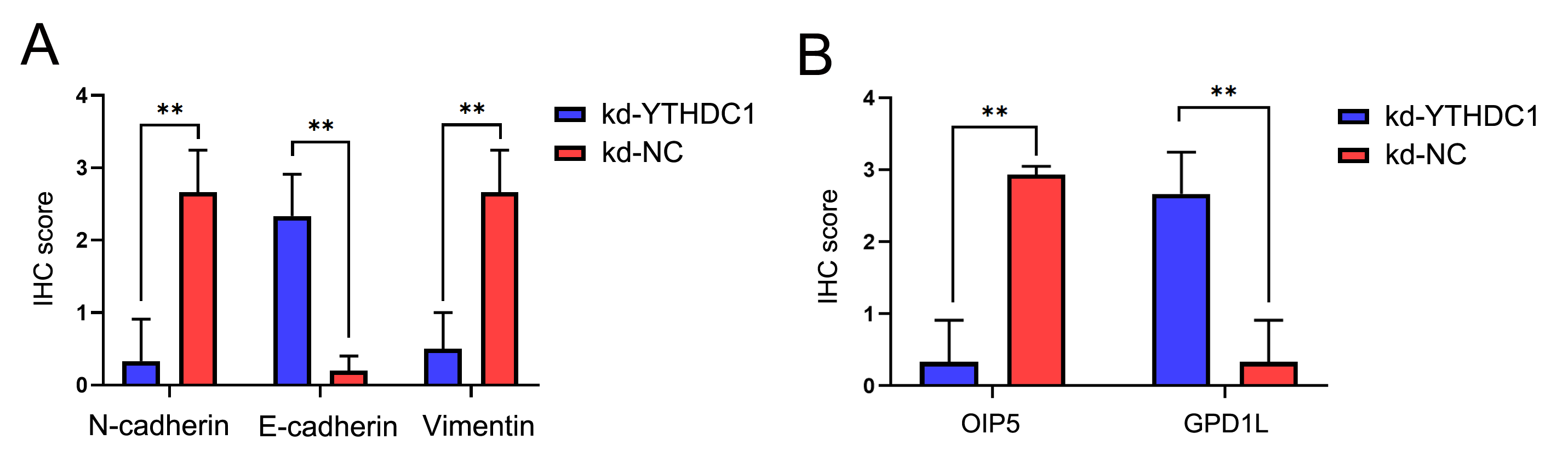

Supplement: Supplementary file 1 — (ZIP 9651 kb) [file 10565_2024_9859_MOESM1_ESM.zip › Supplemental Figure 3.tif]
